# Consistency of a Nonword Repetition Task to Discriminate Children with and without Developmental Language Disorder in Catalan–Spanish and European Portuguese Speaking Children

**DOI:** 10.3390/children8020085

**Published:** 2021-01-26

**Authors:** Nadia Ahufinger, Amy Berglund-Barraza, Anabela Cruz-Santos, Laura Ferinu, Llorenç Andreu, Mònica Sanz-Torrent, Julia L. Evans

**Affiliations:** 1 Estudis de Psicologia i Ciències de l’Educació, Universitat Oberta de Catalunya, 08018 Barcelona, Spain; lferinu@uoc.edu (L.F.); landreub@uoc.edu (L.A.); 2 Department of Speech, Language, Hearing Sciences, University of Texas at Dallas, Richardson, TX 75080, USA; Amy.Berglund@utdallas.edu (A.B.-B.); jle130030@utdallas.edu (J.L.E.); 3 Research Centre in Education, Institute of Education, University of Minho, 4710-057 Braga, Portugal; acs@ie.uminho.pt; 4 Facultat de Psicologia, Universitat de Barcelona, 08035 Barcelona, Spain; monicasanz@ub.edu

**Keywords:** developmental language disorder (DLD), specific language impairment (SLI), nonword repetition, diagnostic markers of DLD/SLI, likelihood ratio, Catalan, European Portuguese

## Abstract

Nonword repetition has been proposed as a diagnostic marker of developmental language disorder (DLD); however, the inconsistency in the ability of nonword repetition tasks (NRT) to identify children with DLD raises significant questions regarding its feasibility as a clinical tool. Research suggests that some of the inconsistency across NRT may be due to differences in the nature of the nonword stimuli. In this study, we compared children’s performance on NRT between two cohorts: the children in the Catalan–Spanish cohort (CS) were bilingual, and the children in the European Portuguese cohort (EP) were monolingual. NRT performance was assessed in both Spanish and Catalan for the bilingual children from Catalonia-Spain and in Portuguese for the monolingual children from Portugal. Results show that although the absolute performance differed across the two cohorts, with NRT performance being lower for the CS, in both Catalan and Spanish, as compared to the EP cohort in both, the cut-points for the likelihood ratios (LH) were similar across the three languages and mirror those previously reported in previous studies. However, the absolute LH ratio values for this study were higher than those reported in prior research due in part to differences in wordlikeness and frequency of the stimuli in the current study. Taken together, the findings from this study show that an NRT consisting of 3-, 4-, and 5-syllable nonwords, which varies in wordlikeness ratings, when presented in a random order accurately identifies and correctly differentiates children with DLD from TD controls the child is bilingual or monolingual.

## 1. Introduction

The ability to repeat nonwords has been associated with early childhood vocabulary knowledge and development, grammar skills, and utterance lengths [1]. Additionally, differences of nonword repetition ability between clinical populations and their age-matched typically developing peers (TD) has been suggested as a useful tool to explore language differences among clinical populations (e.g., developmental language disorder (DLD) [2,3], dyslexia [4], Down’s syndrome [5], etc.). In the case of DLD, nonword repetition tasks (NRT) have been investigated as a method to differentiate TD children from children with DLD, but the results from these studies have been inconclusive. The purpose of this study was to examine the diagnostic consistency of NRT to discriminate between monolingual and/or bilingual children with DLD from TD controls when developed in the native languages of children. More specifically, we compared the precision of language-specific NRT to identify DLD in a group of simultaneous bilingual Catalan–Spanish speaking children with and without DLD to that of a group of monolingual European Portuguese children with and without DLD.

Developmental language disorder (DLD), historically referred to as specific language impairment (SLI), is a neurodevelopmental disorder characterized by normal nonverbal intelligence paired with a persistent inability to master comprehension and production of language in the absence of intellectual or emotional disability, hearing loss, or other medical conditions or syndromes known to cause language disorders [6,7,8]. Historically, the focus of much of the research in DLD has been on determining the degree to which the deficits are specific to the language system or extend to nonlinguistic aspects of cognition. Some researchers historically used the term SLI to refer to children with deficits that were believed to be specific to the “language” system [9], while other researchers used a broader interpretation of the term to denote the presence of both language-based deficits as well as weaknesses in areas that go beyond language [6,7,8]. Consistent with the central tenants of a broad characterization of the disorder, the research community recently shifted to use the term DLD to refer to those children who fall into the broader definition of SLI [10,11,12]. In keeping with this trend, we use the term DLD in this manuscript to refer to this more broadly defined group of children with language-based deficits. Adding support to these domain-general theories of DLD, nonword repetition performance has been consistently shown to be lower in children with DLD than their TD peers [13]. The ability to repeat nonwords relies on numerous underlying factors, such as phonological working memory [3,14,15], auditory processing [16,17], and speech production [18]. Understanding the nature of the underlying cause of poor nonword repetition DLD is seen as a necessary missing piece to understand the underlying cause of language deficits in these children.

Arguments for a phonological working memory basis for the nonword repetition deficits in DLD are founded on Baddeley’s theory of working memory, and more specifically, on the phonological loop portion of his theory [19,20]. The phonological loop’s storage component is particularly implicated in DLD [21,22]. The phonological store is a short-term memory trace of the incoming phonological information that fades over a matter of seconds. It is argued that individuals with DLD have smaller storage capacity in the phonological loop than their TD peers. By using a fast-mapping paradigm, Alt [23] found that children with DLD had difficulty with fast-mapping words that were greater than two syllables long, which she attributed to initial encoding difficulties and decreased storage capacity of the phonological loop. Further evidence for the limited storage capacity comes from studies on nonword repetition in children with DLD, where children with DLD perform relatively similar to their TD peers on words with two syllables, and they perform progressively worse than their peers as the nonwords become longer [24]. These researchers have argued that as the nonword length increases, the amount of storage required to maintain these nonwords in working memory also increases. Thus, the decrease in performance on nonwords of increasing length in DLD is attributed to a lack of adequate phonological storage.

Syllable length is not the only factor shown to influence nonword repetition ability in both DLD and TD populations. Syllabic phonological frequency as well as wordlikeness have both been shown to impact nonword repetition [25,26], with higher phonological frequency and/or high wordlikeness resulting in higher nonword repetition accuracy. These syllabic and word-level influences on nonword repetition performance are unsurprising from Baddeley’s working memory framework. In the model, working memory interacts with long-term, extant knowledge to the effect that a higher frequency nonword is hypothesized to have stronger long-term associative links at the phonological level (in the case of high phonological syllable frequency), or higher word-association links (for high wordlikeness), resulting in greater accuracy for these nonwords due to their decreased demands on memory. Frequency effects such as this have been observed at the word level, with higher frequency words identified quicker during lexical decision tasks as compared to low-frequency words [27,28,29,30].

One aspect of nonword repetition tasks that makes them highly useful for research and diagnostic purposes is that unlike other measures such as sentence comprehension and receptive and expressive vocabulary, NRT does not rely on the underlying structure of the language the task is being administered in, and as such, NRT provides a measure of language ability that is independent of environmental factors such as race and maternal education level [24,31], but one that is not biased by the morphosyntactic structure of the language [32,33,34]. Due to this, in recent years, a multitude of studies have investigated the efficacy of employing NRT to differentiate children with DLD from their TD peers across a wide range of languages including Spanish [32], Norwegian [35], Swedish [36,37], Vietnamese [38], Gulf Arabic [39], Italian [40], Icelandic [41], and Brazilian Portuguese [42]. However, it is important to note that although nonword repetition performance is not influenced by the syntax of a language, it is influenced by the lexicon. Specifically, NRT are sensitive to the underlying phonological, phonetic, syllable frequency, and the degree to which the nonword overlaps with real worlds in the lexicon. Taken together, this suggests that clearly monolingual children should be tested on NRT derived from their native language but also that bilingual children should be tested on NRT derived from all of their native language(s) [40,43,44].

The common deficits in nonword repetition performance in DLD and its cross-linguistic potential have motivated a great deal of research examining the degree to which NRT stimuli can be used to discriminate children with DLD from TD. However, there is large variability in these studies, both with respect to the degree to which performance is poorer for DLD as compared to TD controls *and* in the positive likelihood values reported across studies. Positive likelihood ratios (LH) give the percentage possibility of an individual being a part of a group given a score on a task or test [45], and the larger the LH value, the greater the likelihood the individual is a part of the group. Studies calculating LH values for nonword repetition accuracy report a range of values including small to moderate (LH = 2.78 at or below 70% accuracy [46]; LH = 6.67 at or below 50% accuracy [47]) to moderate-high (LH = 11 at or below 50% accuracy [48]).

A potential factor contributing to the variability across different NRT tasks to discriminate children with DLD from TD children may be differences in the NRT themselves. For example, Ellis Weismer et al. [46] reported data from the Dollaghan and Campbell [49] NRT, which is a version of NRT that consists of a set of 16 nonwords, four at each of four-syllable lengths (1-, 2-, 3-, 4-) that were controlled to all have low wordlikeness ratings. Further, the nonwords are presented in a sequential order beginning with the one-syllable nonwords and progressing to the four-syllable nonwords. In contrast, Girbau and Schwartz [32] examined NRT performance in Spanish–English bilingual children with and without DLD using a NRT task that consisted of 20 nonwords, four at each of five syllable lengths (1-, 2-, 3-, 4-, 5-).

To examine the consistency of NRT as a measure to identify and discriminate children with DLD from children with normal language across languages, in the current study, we compared children’s performance on a NRT task in two ways. First, because there were no NRT in Catalan or European Portuguese, we used the same method to develop two NRT to compare the performance of a group of bilingual Catalan–Spanish speaking children with and without DLD to that of a group of monolingual European Portuguese speaking children. Since the Catalan–Spanish cohort was bilingual, we also compared their performance on a previously developed and published Spanish NRT task.

First, we ask if bilingual versus monolingual status matters when using an NRT task developed from scratch that matches a child’s native language in its ability to differentiate children with DLD from their TD peers who are either simultaneous bilingual (e.g., Catalan–Spanish) or monolingual (e.g., European Portuguese). In line with the current research, we hypothesize that nonword repetition ability will have a high likelihood of predicting which children have DLD and which do not in both language populations. Second, we ask if the syllable length of the nonwords improves NRT’s ability to identify children with DLD over total percent correct. Specifically, we examined whether the likelihood ratio values can accurately identify and differentiate DLD from children having typical language in an individual child. In keeping with the current literature, we hypothesize that because children with DLD may perform similar to their TD peers on shorter nonwords (two syllables), but because their accuracy may decline beyond two syllables, longer nonwords may have better diagnostic accuracy as compared to two-syllable nonwords. Third, we asked to what extent does the degree to which the nonword overlaps with real words in structure (i.e., wordlikeness) influence diagnostic accuracy. Since vocabulary has been shown to influence nonword repetition performance, and previous work consistently shows that performance is poorer for children with DLD on nonwords having low as compared to high wordlikeness, we predict that the wordlikeness of nonwords also will increase the diagnostic accuracy of NRT. Our working hypothesis is that a similar pattern of performance on NRT created in two different languages, using the same method to create the nonword lists, should result in similar LH ratios across the two linguistically different samples, and it would provide valuable data regarding the feasibility of NRT as a diagnostic marker of DLD.

## 2. Materials and Methods

### 2.1. Participants

#### 2.1.1. Catalan–Spanish (CS)

The Catalan–Spanish (CS) cohort consisted of a total of 72 children: 36 children with developmental language disorder (DLD-CS) aged 5;4–15;5 and 36 typically developing children (TD-CS) with normal language aged 5;7–16;2 (A portion of the children of the Catalan–Spanish cohort were included in Ahufinger et al. [50]). There were a total of 20 girls (28%) and 52 boys (72%). The children with DLD-CS were pairwise matched to TD-CS controls based on age (+/−3 months) and sex at the time of the study. All the children were native simultaneous bilingual speakers of Catalan and Spanish that were exposed to both languages from birth. (Catalan is one of the 5 co-official languages that coexist in Spain. It is only spoken in a region called *Països Catalans*/Catalan countries formed by Catalonia, Valencia, Balearic Islands, and Northern Catalonia). According to the parental survey, all families of the present study reported that their children speak Catalan and Spanish. All children that participated in the present study were from different areas of Barcelona and the surrounding area (metropolitan area of Barcelona) where Central Catalan is spoken. In 2018, 52.7% of Barcelona citizens claimed that their initial language was Spanish, and 31.5% was Catalan [51]. In the school system, Catalan is the primary language of instruction. Later, in primary school, children receive 2–3 h of Spanish classes per week, while the rest of the subjects are taught in Catalan [52,53]. According to Alarcón and Garzón [54], children in Barcelona are equally proficient in both Spanish and Catalan, although the use of Spanish is more popular. For further information about Catalan and Spanish bilingualism and DLD, see Sanz-Torrent, Badia, and Serra [55] and Sanz-Torrent et al. [56]. As such, the Catalonian children in this study are considered simultaneously bilingual. The children with DLD-CS were selected with the collaboration of different institutions, organizations, and schools as Catalan Center of Resources for Hearing-Impaired People (CREDA), members of the Catalan service for school counseling and guidance (EAP) and Catalan Association of Specific Language Impairment (ATELCA). All the families who agreed to participate in the study were asked to sign an informed consent form. A final report containing the results of all the tests administered to the children was given to the family as a token of gratitude for their commitment and contribution to the study.

##### Catalan–Spanish Inclusion/Exclusion Criteria

The inclusion/exclusion criteria were defined following the DLD diagnostic criteria recommended by a Spanish expert committee that reached a consensus in 2015 [57]. All the children identified were assessed by two trained researchers to confirm the diagnostic criteria. The diagnostic inclusion/exclusion criteria for children with DLD-CS in the present study were as follows: (a) Catalan–Spanish Bilingual; (b) a nonverbal intellectual quotient (NVIQ) > 75 (Kaufmann Brief Intelligence Test Matrices section, K-BIT [58]); (c) a score of –1.25 SD or greater on one of the three scales of the Clinical Evaluation of Language Fundamentals—Fourth Edition, Spanish (CELF-4; [59]): core language, expressive language, and receptive language; (d) normal hearing at 500, 1000, 2000, and 4000 Hz at 20 dB based on the American Speech-Language Hearing Association (ASHA) 1997 guidelines for hearing screening [60]; (e) normal or corrected-to-normal vision; (f) normal oral and speech motor abilities; and (g) absence of other medical or neurological conditions. The inclusion/exclusion criteria for the group of TD children were as follows: (a) Catalan–Spanish Bilingual; (b) a NVIQ > 85 (K-BIT Mat [58]); (c) standardized language scores within the normal range in the core language, expressive language, and receptive language CELF-4 scales; and (d) absence of a prior history of speech or psychological therapy (see Table 1). All children were classified as either TD or DLD based on standard clinical practice in Catalonia, which is based on the Spanish version of the standardized test where all stimuli are presented in Spanish, but if children answered correctly in Catalan, they were given credit for their answer. There currently are no normative language tests for the Catalan language. From 79 children with language difficulties that were initially screened, 43 were excluded for failure to meet the inclusion/exclusion criteria.

#### 2.1.2. European Portuguese (EP)

The European Portuguese cohort consisted of a total of 150 children; 75 children with developmental language disorder (DLD-EP) aged 7;0–11;11, and 75 European Portuguese speaking typically developing (TD-EP) children with normal language aged 7;0–11;11. There were a total of 42 girls (28%) and 108 boys (72%). The children with DLD-EP were pairwise matched to typical controls based on age (+/−6 months) and sex from the same classroom and school grade.

Prior to participant recruitment, permission to perform this study in the state-funded schools in the North of Portugal was requested from the Board of Education—North Division (DREN, Direcção Regional de Educação do Norte). First, the experimenter visited each of the 17 schools and presented the aims of the study to the school administrator, who gave his formal consent for the experimenter to contact each classroom teacher in order to gather the required information. Then, classroom regular and special education teachers were asked to identify children in the 7-, 8-, 9-, 10-, and 11-year-old age groups who had language problems (i.e., oral comprehension, oral production, reading problems, language delay, etc.) that might be potential DLD participants. All this identification process was based on existing assessments (e.g., portfolios, checklists, inventories) at the school at the time of the study. Once a list of children with potential DLD-EP was obtained, a written consent form for soliciting participation in this study was forwarded to the parents of the children in the target age ranges. Once the participants with DLD-EP in a given school were identified and parental consent was returned to the experimenter; then, typical developed controls were identified using the same procedures.

##### European Portuguese Inclusion/Exclusion Criteria

The inclusion/exclusion criteria were defined following the Portuguese National criteria. The diagnostic inclusion/exclusion criteria for children with DLD-EP in the present study were (a) Monolingual European Portuguese speaking—the only spoken language in the home setting; (b) a nonverbal intellectual quotient (NVIQ) > 80 assessed by school psychologists with the Wechsler test standardized for the European Portuguese population (WISC-III; [61]); (c) language impairment; (d) normal hearing; (e) normal or corrected-to-normal vision; (f) normal oral and speech motor abilities; and (g) absence of other medical or neurological conditions. Criteria a, c, d, e, f, and g were based on existing classroom observations, interviews, informal developmental checklists, portfolios, school reports, medical/clinical reports, and formal academic assessments provided by parents and teachers. At the time of data collection, there were no standardized language assessment measures in European Portuguese. The inclusion/exclusion criteria for the group of TD children were as follows: (a) Monolingual European Portuguese speaking—the only spoken language in the home setting; (b) a nonverbal intellectual quotient (NVIQ) > 80 assessed by school psychologists with WISC-III [61]; (c) normal language abilities; and (d) absence of a prior history of speech or psychological therapy. Criteria a, c, and d were based on interviews, portfolios, school reports, medical/clinical reports, and formal academic assessments provided by parents and teachers. The children all attended state-funded primary and intermediate grades (2nd to 5th grade) in schools located in a range of urban, suburban, and rural areas in two districts in the North of Portugal. A total of 179 children were initially identified for the study; 29 were subsequently excluded for failure to meet the inclusion/exclusion criteria and/or low NVIQ.

### 2.2. Nonword Repetition Task

#### 2.2.1. Catalan Nonword Repetition Task (NRT-Cat)

Since there was no NRT task in Catalan (NRT-Cat), we developed one for the purpose of the current study. Based on a Graf Estes, Evans, and Else-Quest [3] meta-analysis showing that the Children’s Test of Nonword Repetition (CNRep; [62]) has larger effect sizes in detecting DLD, we designed the task to mirror that of the CNRep ([62]) with respect to the total number of nonwords and distribution of high- and low wordlikeness of the nonwords. Additionally, the NRT-Cat was developed taking into account the original criteria established by Gathercole et al. [62]: “minimal articulatory output demands”, “dominant syllable stress patterns”, and “phoneme sequences within each non-word were all phonotactically and prosodically legal” (p. 106) for the English language.

##### Syllable Length

To develop the NRT-Cat, we first generated lists of words selected from a large corpus of children’s songs and books as well as from spontaneous language samples. Verbs, diphthongs, and words with orthographic stress were not excluded from the list. We generated a list of 100 Catalan nonwords with different syllable length (from 2 to 6 syllables; each syllable length had 20 nonwords) taking into account characteristics of Catalan language such as the number of syllables, complexity, and prosody. The syllable stress pattern for Catalan was analyzed for development of the nonwords. It was taken into account that for the Catalan language, almost all of the words that finish in a consonant have the stressed pattern in the last syllable (i.e., perfil/capital); multi-syllabic words that finish with vowels usually have the stressed pattern in the penultimate syllable (i.e., divorci/capsa). All the words in Catalan that have the stressed syllable in the antepenultimate position have orthographic accent (e.g., música), but these words were not included.

All of the Catalan nonwords began with consonants that occur regularly at the word initial position in Catalan (see Table A1); nonwords could finish with a consonant or vowel; nonwords contained Catalan regular stress patterns (non-orthographic stress patterns as described in the previous paragraph), diphthongs, and orthographic stress patterns were not included in the nonwords. Similar to the CNRep’s original criteria [62], the phoneme sequences in each nonword conform to the phonotactic rules of Catalan. Additionally, similar to the methodological criteria outlined in Gathercole et al. [62], consonants that occur naturally in Catalan and double consonants clusters naturally occurring in Catalan were included to build the Catalan nonwords (e.g., *Galmet*, *Becra*, *Marlut, Palimatrenc*, *tropa*, etc.).

##### Wordlikeness

The degree to which a nonword “sounds like” a real word in a language has been shown to influence nonword repetition ability in children with and without DLD [3]. Moreover, it has been argued that “phonotactic knowledge of the possible/probable sequences of sounds within a language might be derived directly from the mental lexicon, depending on their similarity to known words” ([63] p. 568). We used the same procedure as Gathercole et al. [62] to determine the wordlikeness of each of the nonwords. A native female speaker of the Central Catalan dialect was recorded to create a digital recording of the list of 100 nonwords in a randomized order in 5 sets of 20 nonwords. Each set had 10 single words (CVC) and 10 consonant cluster words (CCV/CCVC). These words were presented to a group of 20 native Catalan-speaking adults (male and female). They were asked to rate how “wordlike” each of the nonwords auditory presented was according to the similarity they have with Catalan words. They had to do it trying to answer the following question: Would it be possible that this word is in the Catalan language? We used a scale that ranged from 1 (“It has very few possibilities”) to 5 (“It has excellent possibilities”). The adults rated each nonword immediately upon hearing it on a scoring sheet. Then, the data were analyzed to calculate means of the 100 nonwords. Words were listed in rank order for each syllable length, with nonwords receiving the highest wordlikeness ratings ranked at the top and nonwords with the lowest wordlikeness ranked at the bottom. Next, the four nonwords from the bottom of the ranking that received the lowest mean wordlikeness ratings were chosen for each of 2-, 3-, 4-, 5-, and 6-syllable lengths, creating a list of 20 low wordlikeness nonwords. Likewise, the four nonwords at the top of the ranking that received the highest mean wordlikeness ratings for each of the 2- 3-, 4-, 5-, and 6-syllable lengths were selected, creating a list of 20 high wordlikeness nonwords. For the final list for each syllable length, four of the nonwords contained single consonants and four contained one or more consonant clusters. All the words had the same number of phonemes at each of the syllable lengths. A complete list of the 40 nonwords by high and low Wordlikeness for 2-, 3-, 4-, 5-, and 6-syllable lengths is shown in Table 2. Their phonetic transcriptions in International Phonetic Transcription (IPA) are shown in Table A1.

#### 2.2.2. Spanish Nonword Repetition Task (NRT-Span)

The version of the Spanish nonword repetition task used in this study was a prior published version developed by Aguado [64]), which consists of a set of 80 nonwords that range from 2 to 5 syllables in length. The nonwords were also classified according to their overall frequency calculated at the syllable level (high frequency, low frequency) based on the database of Alameda and Cuetos [65]. The final NRT stimuli were divided into two lists (high frequency or low frequency) with each list having four groups of ten nonwords ranging from 2 to 5 syllables in length (10 words at each length in each list). Both nonword lists are controlled to have an equal number of syllables of nonwords, syllable structure, stress pattern, and order in which the syllables with their different structures were placed. Two phoneme consonant clusters were included. The task is based on children listening to a series of nonwords one by one, temporarily retaining the phonological information of these and then producing them verbally.

#### 2.2.3. European Portuguese Nonword Repetition Task (NRT-EPort)

##### Syllable Length

Similar to Catalan, because there was no European Portuguese NRT, we developed one for the purpose of the current study using the same steps in developing the stimuli that we used for the Catalan NRT. For the NRT-EPort, we first generated lists of words selected from a large corpus of European Portuguese derived from interview-based corpora [66,67] and children’s songs, children’s books, grammar books, and European Portuguese language development studies [68,69,70,71,72,73]. Then, these real words were classified according to the number of syllables, articulatory complexity, and prosody. Verbs, diphthongs, and words with orthographic stress were not excluded from the list. From the remaining set of words, a set of 100 nonwords were generated from these real words that differed in syllable length (from 2 to 6 syllables) and contained naturally occurring European Portuguese stress patterns [67,69]. Syllable stress patterns in European Portuguese were analyzed for development of the nonwords. The stress is always on the second to last syllable regardless of syllable length. Some examples are 2-syllable words (bola/ball); 3-syllable words (panela/pot); 4-syllable words (fortaleza/fortress); 5-syllable words (psicologia/ psychology); 6-syllable words (corajosamente/bravely).

The result was a set of 2-, 3-, 4-, 5-, and 6-syllable nonwords (20 per syllable length). All nonwords begin with consonants, and the consonants /r/, /l/, /∫/, /m/, and the vowels /ɔ/, /u/, /ɐ̃/, /ɐ/, /w/, /i/ never occurred in the final position of the nonwords. Similar to the CNRep’s original criteria [62], two phoneme consonant clusters that occur within European Portuguese were included (e.g., *branco*, *plasma*, *grito*, *tropai*, etc.).

##### Wordlikeness

The same procedure as the Catalan version of the NRT was followed to derived wordlikeness rating for the nonwords in European Portuguese NRT. For each syllable length, four of the nonwords contained single consonants, and four contained one or more consonant clusters. All the words had the same number of phonemes for each of the syllable lengths. The nonwords had no plurals or Portuguese complex morpheme endings (e.g., mente). A complete list of the 40 European Portuguese nonwords, by high and low wordlikeness for 2-, 3-, 4-, five -, and 6-syllable lengths, is shown in Table 3. Their phonetic transcriptions in International Phonetic Transcription (IPA) are shown in Table A2.

### 2.3. Administration and Scoring of NRT-Cat, NRT-Spa, and NRT-EPort

For the Catalan and European Portuguese versions of the NRT, there was a fixed randomized order across all the nonwords such that the listener could not predict the syllable length and wordlikeness. For the Spanish version of the task, the 40 nonwords of each list (high nonword frequency and low nonword frequency) were presented sequentially in accordance to syllable length (i.e., all 2-syllable nonwords followed by all 3-syllable nonwords, etc.). Half of the sample was administered first in the high-frequency list and then the low-frequency list, and with the other half, the order was inverted.

To control for possible presentation effects, a digital recording of each of the NRTs was created by an adult female native speaker for each language. The female adult said each nonword aloud at the rate of one nonword every three seconds. The child’s task was to repeat each nonword immediately. For each NRT task, children heard two practice items (e.g., *ticopo* and *mastruca* for Catalan and Spanish, and *banata* and *mencolate* for European Portuguese), and these were repeated until the child understood the task completely. Each child heard the following instructions as in other studies [62]:

*“I would like you to play a game with me! When I switch on the disc in a minute (point to the recorder), you will hear a funny made-up word, a word that does not exist. I would like you to repeat the funny word back to me as soon as you have heard it. Did you understand? So, if the made-up word you heard was banata, you should say banata back to me. Let’s try that now, ok?”.*

Catalan: *“M’agradaria molt que juguéssim junts/es! Ara quan encengui l’altaveu escoltaràs unes paraules divertides que estan inventades, són paraules que no existeixen. El que has de fer és repetir la cada paraula divertida just després d’escoltar-la. Ho has entès? Per exemple, si la paraula inventada que escoltes fos plàtan, tu has de dir plàtan. Anem a intentar-ho, d’acord?”*

Spanish: *“¡Me gustaría mucho que jugáramos juntos/as! Ahora cuando encienda el altavoz escucharás unas palabras divertidas que están inventadas, son palabras que no existen. Lo que tienes que hacer es repetir cada palabra divertida justo después de escucharla. ¿Lo has entendido? Por ejemplo, si la palabra inventada que escuchas fuera plátano, tú debes decir plátano. Vamos a intentarlo, ¿de acuerdo?”*

Portuguese: *“Vamos jogar um jogo! Quando eu ligar o gravador vais ouvir uma palavra inventada, que não existe, mas muito engraçada. Quero que repitas a palavra tal como a ouves, está bem? Vamos experimentar agora. Quando ouvires a palavra banata vais dizer banata tal e qual como a ouviste, está bem?”*

All the children in both of the language cohorts were able to perform the task following the practice items.

### 2.4. Scoring of the NRT-Cat, NRT-Spa, and NRT-EPort

For each NRT task, an adult experimenter scored the child’s repetition attempt as it was produced, using a simple binary scoring procedure in which a correct repetition was scored as 1 (judged by the experimenter to be phonologically accurate), and an incorrect attempt was scored as 0 if the experimenter judged that the child had produced a sound that differed from the target nonword by one or more phonemes. Children’s responses were also recorded and subsequently rescored from the recording a second time to ensure the accuracy of scoring. Correct and incorrect responses were annotated on the answer sheet by the experimenter. The total number of nonwords spoken correctly was calculated for each child.

### 2.5. Reliability: Scoring of the NRT-Cat, NRT-Spa, and NRT-EPort

For the Catalan NRT (NRT-Cat), a total of 28% (*n* = 20) of the children’s responses were randomly identified from the sample to test reliability (DLD-CS = 10; TD-CS = 10). This reanalysis was performed by two trained independent Catalan–Spanish researchers. The scoring–rescoring agreement was 92.75% for the NRT-Cat Test for children with DLD-CS and was 97.5% for the TD-CS controls.

For the Spanish NRT (NRT-Spa), a total of 28.5% (*n* = 20) of the children’s responses were randomly identified from the sample to test the reliability (DLD-CS = 10; TD-CS = 10). This reanalysis was performed by two trained independent Catalan–Spanish researchers. Scoring–rescoring agreement was 94.6% for the NRT-Cat Test for children with DLD-CS and 96.9% for the TD-CS controls.

For the European Portuguese NRT (NRT-Port), a total of 20% (*n* = 30) of the children’s responses were randomly identified from the sample to test reliability (DLD-EP = 18; TD-EP = 12). This reanalysis was performed by two trained independent European Portuguese graduate adults. The scoring–rescoring agreement was 87.5% for the NRT-EPort Test for children with DLD-EP, and it was 92.5% for the TD-EP controls.

## 3. Results

### 3.1. Group Differences on the NRT

#### 3.1.1. Syllable Length

The means and standard deviations on the NRT (NRT-Cat) for total percentage of words produced correctly (TPWC) for each syllable length are presented in Table 4 for the Catalan–Spanish and European Portuguese cohorts.

##### Catalan–Spanish Cohort

Looking first at the Catalan version of the NRT, a univariate analysis of variance, controlling for age (ANCOVA) was first conducted comparing the overall nonword repetition performance for the Catalan nonwords for the DLD-CS and TD-CS groups. The analysis revealed a significant main effect of group *F*(1, 71) = 150.86, *p* < 0.001, partial *η*^2^ = 0.68, power = 1.0, where the DLD-CS group’s performance was significantly poorer than that of the TD-CS controls. A follow-up 2 × 5 Repeated Measures ANCOVA was conducted with group (DLD-CS, TD-CS) × syllable length (2-, 3-, 4-, 5-, 6-syllable) as the between and within subject variables, and age as the covariate, to examine differences in the groups’ performance for each syllable length. Mauchly’s test indicated that the assumption of sphericity was violated; therefore, degrees of freedom were corrected using Greenhouse–Geisser estimates of sphericity [74]. The analysis revealed a significant main effect of group *F*(1, 69) = 152.78, *p* < 0.001, partial *η*^2^ = 0.68, power = 1.00; and significant main effect for syllable length, *F*(3.36, 231.89) = 13.29, *p* < 001, partial *η*^2^ = 0.16, power = 1.00, and a significant length × group interaction *F*(3.36, 231.89) = 23.65, *p* < 0.001, partial *η*^2^ = 0.25, power = 1.00. Follow-up analyses of covariance (ANCOVA) were conducted for each of the syllable length with age as a covariate to examine group differences at each of the six syllable lengths. The analysis revealed that the TD-CS performance was significantly better than that of the children with DLD-CS for all syllable lengths: 2-syllable *F*(1, 71) = 51.32, *p* < 0.001, partial *η*^2^ = 0.42, power = 1.0; 3-syllable *F*(1, 71) = 40.13, *p* < 0.001, partial *η*^2^ = 0.36, power = 1.0; 4-syllable, *F*(1, 71) = 65.30, *p* < 0.001, partial *η*^2^ = 0.48, power = 1.0; 5-syllable, *F*(1, 71) = 121.47, *p* < 0.001, partial *η*^2^ = 0.63, power = 1.0; 6-syllable, *F*(1, 71) = 150.06, *p* < 0.001, partial *η*^2^ = 0.68, power = 1.0. The DLD-CS group performed worse than the TD-CS group at repeating each nonword across the syllable lengths (2-, 3-, 4-, 5-, 6- syllable). This finding illustrates that a higher percentage of correct nonwords are achieved at the lowest lengths, decreasing to a lower percentage of correct nonwords as the syllable length also increases (from 2 syllables to 6 length) for both groups.

For the Spanish version of the NRT (NRT-Spa), a univariate analysis of variance, controlling for age (ANCOVA) was first conducted comparing overall nonword repetition performance for the Spanish nonwords for the DLD-CS and TD-CS groups (Two of the DLD-CS participants did not complete the Spanish NRT). The analysis revealed a significant main effect of group *F*(1, 69) = 130.8, *p* < 0.001, partial *η*^2^ = 0.66, power = 1.0, where the DLD-CS group’s performance in repeating the Spanish nonwords was significantly poorer than that of the TD-CS controls. A follow-up 2 × 5 Repeated Measures ANCOVA was conducted with group (DLD, TD) × syllable length (2-, 3-, 4-, 5-syllable) as the between and within variables, and age as the covariate, to examine differences in the groups’ performance for each syllable length. Mauchly’s test indicated that the assumption of sphericity was violated; therefore, degrees of freedom were corrected using Greenhouse–Geisser estimates of sphericity [74]. The analysis revealed a significant main effect of group *F*(1, 67) = 127.4, *p* < 0.001, partial *η*^2^ = 0.65, power = 1.00; and significant main effect for syllable length, *F*(2.47, 166.0) = 11.2, *p* < 0.001, partial *η*^2^ = 0.14, power = 0.99, and a significant length × group interaction *F*(2.47, 166.0) = 58.4, *p* < 0.001, partial *η*^2^ = 0.46, power = 1.00. Follow-up analyses of covariance (ANCOVA) were conducted for each of the syllable length, with age as a covariate to examine group differences at each of the six syllable lengths for the NRT-Spa. The analysis revealed that the TD-CS performance was significantly better than that of the children with DLD-CS for all syllable lengths: 2-syllable *F*(1,67) = 28.9, *p* < 0.001, partial *η*^2^ = 0.30, power = 1.0; 3-syllable *F*(1, 67) = 40.93, *p* < 0.001, partial *η*^2^ = 0.37, power = 1.0; 4-syllable, *F*(1, 67) = 110.9, *p* < 0.001, partial *η*^2^ = 0.62, power = 1.0; 5-syllable, *F*(1, 67) = 166.3, *p* < 0.001, partial *η*^2^ = 0.71, power = 1.0. The DLD-CS group performed worse than the TD-CS group at repeating each Spanish nonword across the syllable lengths (2-, 3-, 4-, 5-syllable). This finding illustrates that a higher percentage of correct nonwords is achieved at the lowest lengths, decreasing to a lower percentage of correct nonwords as the syllable length also increases (from 2-syllable to 5-syllable length) for both groups.

Finally, we compared the performance on the NRT-Cat and NRT-Spa tasks for the DLD-CS to determine if the performance for the DLD-CS cohort differed depending upon which language their phonological working memory was assessed. A Repeated Measures ANCOVA was conducted comparing total percentage correct for the Catalan and Spanish versions of the task (NRT-Cat and NRT-Spa) as the within variables, and age as the covariate, to examine differences in the DLD groups’ performance for the NRT performance in Catalan as compared to Spanish. Mauchly’s test indicated that the assumption of sphericity was not violated; therefore, degrees of freedom were not corrected using Greenhouse–Geisser estimates of sphericity [74]. The analysis revealed that there was not a significant main effect of language *F*(1, 32) = 0.54, *p* = 0.46, partial *η*^2^ = 0.01, power = 0.11, indicating that the DLD-CS group’s performance in repeating the Spanish nonwords did not differ from their performance repeating the Catalan nonwords. We next compared the performance of the TD-CS group on the NRT-Spa and NRT-Cat tasks. For the TD-CS, Mauchly’s test indicated that the assumption of sphericity was not violated, and a Repeated Measures ANCOVA comparing total percentage correct for the NRT-Cat and NRT-Spa for the TD-CS controls revealed no difference in the TD-CS ability to repeat the Catalan and Spanish nonwords *F*(1, 34) = 0.29, *p* = 0.59, partial *η*^2^ = 0.00, power = 0.08.

##### European Portuguese

The means and standard deviations for nonword repetition for percentage of words correct (PWC) for the total task and each syllable length are presented in Table 4 for the DLD-EP and TD-EP groups. Univariate analysis of variance, controlling for age (ANCOVA), was used to assess group differences in total percentage of words correct (TPWC) for the DLD-EP and TD-EP groups. The analysis revealed a significant effect of group *F*(1, 149) = 232.00, *p* < 0.001, partial *η*^2^ = 0.61, power = 1.0, where the DLD-EP group performed significantly worse in repeating the nonwords as compared to the TD-EP controls. A 2 × 5 Repeated Measures ANCOVA was conducted with group (DLD, TD) × syllable length (2-, 3-, 4-, 5-, 6-syllable) as the between and within variables, and age as the covariate, to test for group differences in nonword repetition at each syllable length. Mauchly’s test indicated that the assumption of sphericity had been violated; therefore, degrees of freedom were corrected using Greenhouse–Geisser estimates of sphericity [74]. The analysis revealed a significant main effect for group *F*(1, 147) = 232.00, *p* < 0.001, partial *η*^2^ = 0.61, power = 1.0, a significant effect of syllable length, *F*(3.74, 550.01) = 5.72, *p* < 001, partial *η*^2^ = 0.03, power = 0.97, and a significant length × group interaction *F* (3.74, 550.01) = 25.81, *p* < 0.001, partial *η*^2^ = 0.14, power = 1.00. Follow-up analyses of covariance (ANCOVA) were conducted for each of the syllable lengths, with age as a covariate. Results indicated that the TD-EP’s nonword repetition was better at all lengths as compared to the DLD-EP children: 2-syllable *F*(1, 149) = 89.51, *p* < 0.001, partial *η*^2^ = 0.37, power = 1.0; 3-syllable *F*(1, 149) = 86.04, *p* < 0.001, partial *η*^2^ = 0.36, power = 1.0; 4-syllable, *F*(1, 149) = 94.30, *p* < 0.001, partial *η*^2^ = 0.39, power = 1.0; 5-syllable, *F*(1, 149) = 58.11, *p*< 0.001, partial *η*^2^ = 0.28, power = 1.0; 6-syllable, *F*(1, 149) = 243.51, *p* < 0.001, partial *η*^2^ = 0.61, power = 1.0. The results indicated that nonword repetition accuracy was poorer for both the DLD-EP as compared to TD-EP controls both for total words correct and at each syllable length.

#### 3.1.2. Wordlikeness/Nonword Frequency

The means and standard deviations for nonword repetition for total percentage of words produced correctly (TPWC) for nonwords having high and low wordlikeness ratings are presented in Table 5 for the Catalan–Spanish and European Portuguese cohorts.

##### Catalan–Spanish Cohort

Looking first at the NRT-Cat, for the DLD-CS, the total percentage of correct nonword repetition of the Catalan nonwords having high wordlikeness ratings was 52.0% (18.8), and for nonwords having low wordlikeness ratings, it was 44.03% (19.0). For the TD controls, the total percentage of correct nonword repetition for nonwords having high wordlikeness ratings was 87.6% (9.3), and for nonwords having low wordlikeness ratings, it was 88.6% (9.4). A 2 × 2 Repeated Measures ANCOVA was conducted with group (DLD, TD) × wordlikeness (High, Low) as the between and within variables, and age as the covariate, to test for group differences based on wordlikeness. Mauchly’s test indicated that the assumption of sphericity had not been violated; therefore, degrees of freedom were not corrected using Greenhouse–Geisser estimates of sphericity [74]. The analysis revealed a significant main effect for group *F*(1, 69) = 149.6, *p* < 0.001, partial *η*^2^ = 0.68, power = 1.0, a significant wordlikeness × group interaction *F*(1, 69) = 12.1, *p* < 0.001, partial *η*^2^ = 0.15, power = 0.93, but no main effect of wordlikeness, *F*(1, 69) = 1.22, *p* = 0.273, partial *η*^2^ = 0.01, power = 0.19. The results indicated that the nonword repetition performance based on total words correct was greater for the TD-CS controls as compared to the children with DLD-CS regardless of the wordlikeness ratings of the nonwords. Furthermore, the nonword repetition performance for the children with DLD-CS was significantly poorer as compared to that of the TD-CS controls for nonwords having low wordlikeness as compared to nonwords having high wordlikeness ratings.

Looking next at the NRT-Spa, a 2 × 2 Repeated Measures ANCOVA was conducted with group (DLD-CS, TD-CS) × nonword frequency (High, Low) as the between and within variables, and age as the covariate, to test for group differences based on wordlikeness. Mauchly’s test indicated that the assumption of sphericity had not been violated; therefore, degrees of freedom were not corrected using Greenhouse–Geisser estimates of sphericity [74]. The analysis revealed a significant main effect for group *F*(1, 67) = 130.84, *p* < 0.001, partial *η*^2^ = 0.54, power = 1.0, a significant nonword frequency × group interaction *F*(1, 67) = 7.3, *p* < 0.01, partial *η*^2^ = 0.09, power = 0.76, and a significant main effect of nonword frequency, *F*(1, 69) = 10.29, *p* < 0.01, partial *η*^2^ = 0.13, power = 0.88. The results indicated that for the NRT-Spa, nonword repetition performance based on total words correct was greater for the TD-CS controls as compared to the children with DLD-CS regardless of the nonword frequency ratings of the nonwords. In contrast to the NRT-Cat, nonword repetition performance for the Spanish nonwords for the children with DLD-CS was no different from that of the TD-CS controls for nonwords having low nonword frequency as compared to nonwords having high nonword frequency ratings.

##### European Portuguese

For the DLD-EP, the total percentage of correct nonword repetition for nonwords having high wordlikeness ratings was 75.1% (11.99), and for nonwords having low wordlikeness ratings, it was 67.0% (15.1). For the TD controls, the total percentage of correct nonword repetition for nonwords having high wordlikeness ratings was 94.8% (5.4), and for nonwords having low wordlikeness ratings, it was 91.3% (7.6). A 2 × 2 Repeated Measures ANCOVA was conducted with group (DLD, TD) × wordlikeness (High, Low) as the between and within variables, and age as the covariate, to test for group differences based on wordlikeness. Mauchly’s test indicated that the assumption of sphericity had not been violated; therefore, degrees of freedom were not corrected using Greenhouse–Geisser estimates of sphericity [74]. The analysis revealed a significant main effect for group *F*(1, 147) = 232.00, *p* < 0.001, partial *η*^2^ = 0.61, power = 1.0, a significant wordlikeness × group interaction *F*(1, 147) = 10.14, *p* < 0.001, partial *η*^2^ = 0.06, power = 0.88, but no effect of wordlikeness, *F*(1, 147) = 3.04, *p* = 0.083, partial *η*^2^ = 0.02, power = 0.40. Similar to the Catalan–Spanish cohort, the results indicated that nonword repetition accuracy based on total words correct was greater for the TD-EP controls as compared to the children with DLD-EP regardless of the wordlikeness ratings of the nonwords. Furthermore, the nonword repetition accuracy for the children with DLD-EP was significantly poorer as compared to that of the TD-EP controls for nonwords having low wordlikeness as compared to nonwords having high wordlikeness ratings.

##### Differences between the Catalan–Spanish and European Portuguese Speaking Cohorts

To examine potential differences in performance for the Catalan–Spanish and European Portuguese speaking children, we first directly compared the performance for the DLD-CS children with that of the DLD-EP children first on the NRT-Cat and then NRT-Spa. A univariate analysis of variance was conducted comparing the overall nonword repetition performance for the DLD-CS in NRT-Cat and DLD-EP groups. The analysis revealed a significant main effect of group *F*(1, 110) = 62.5, *p* < 0.001, partial *η*^2^ = 0.36, power = 1.0, where the DLD-CS group’s performance was significantly poorer than that of the EP-DLD group. To determine if the same pattern held for the CS cohort in Spanish, a second univariate analysis of variance was conducted comparing the overall nonword repetition performance on the NRT-Spa for the DLD-CS and DLD-EP group. The analysis again revealed a significant main effect of group *F*(1, 108) = 30.3, *p* < 0.001, partial *η*^2^ = 0.22, power = 1.0, where the DLD-CS group’s performance on the NRT-Spa was also significantly poorer than that of the DLD-EP group.

We next compared the performance for the TD-CS children with that of the TD-EP children first on the NRT-Cat and then NRT-Spa. A univariate analysis of variance was conducted comparing overall nonword repetition performance for the TD-CS in NRT-Cat and TD-EP group. The analysis revealed a significant main effect of group *F*(1, 110) = 16.0, *p* < 0.001, partial *η*^2^ = 0.12, power = 0.98, where the TD-CS group’s performance was significantly poorer than that of the TD-EP group. To determine if the same pattern held in for the CS cohort in Spanish, a second univariate analysis of variance was conducted comparing overall nonword repetition performance on the NRT-Spa for the TD-CS and TD-EP groups. The analysis again revealed a significant main effect of group *F*(1, 110) = 12.0, *p* < 0.01, partial *η*^2^ = 0.10, power = 0.93, where the TD-CS group’s performance on the NRT-Spa was also significantly poorer than that of the TD-EPgroup.

In sum, for both the DLD and TD children in the Catalan–Spanish-speaking cohort, nonword repetition ability was poorer as compared to that of the European Portuguese-speaking cohort both when compared in Catalan and Spanish.

### 3.2. Use of NRT to Rule In/Rule Out Developmental Language Disorder

The above analysis replicates prior work showing that poor performance for children with DLD on NRT having low wordlikeness (i.e., [49]) is well documented (i.e., [46]). However, one question is related to the *diagnostic accuracy* of children’s performance on NRT that vary BOTH by syllable length AND wordlikeness to identify the presence of DLD in Catalan–Spanish and European Portuguese-speaking children.

#### 3.2.1. Total Percentage of Words Correct (TOT PWC)

In this study, we calculated the likelihood ratios (LH) for children’s performance on the NRT to assess its diagnostic accuracy. Ultimately, the value of a diagnostic test will depend upon its ability to alter a pre-test probability of a target condition into a post-test probability that will influence a clinical management decision. The positive LH ratio is the ratio of the proportion of patients who have the target condition and test positive to the proportion of patients without the target condition who also test positive. In conveying the meaning of diagnostic accuracy to clinicians, research suggests that LH ratios are more interpretable to clinicians and enable more appropriate interpretation of tests as compared to sensitivity and specificity measures. Specifically, general practitioners when asked to estimate the probability of a disease in a given patient give the most appropriate estimation of test performance when provided LHs as compared to sensitivity/specificity values as compared to LH ratios [75].

Likelihood ratio (LH) analyses were conducted using the presence/absence of DLD based on the gold standard classification for the diagnosis of DLD in each cohort to determine whether Catalan–Spanish and European Portuguese-speaking children’s ability to repeat nonwords serves as a screening tool to detect and diagnose children with DLD in each cohort [76,77]. To determine the LH ratio for a positive result based on total percentage of words correct at test on the NRT (TOT-PWC), the true positive rate (proportion of children with DLD with a total NRT (TOT-PWC) at or below *x-determined* cutoff) was divided by the false positive rate (proportion of TD children with total NRT at or below *x-determined* cutoff) for each cohort. We used Haynes et al. [77] criteria to classify a positive test (i.e., accurately ruling in the disorder), which includes the following: (1) “High” as defined as LH ratio of 20 or higher having a probability of 95% or greater that the disorder is present, (2) “Intermediate High” defined as an LH ratio between 1 and 20, and (3) “Indeterminate” defined as an LH close to 1.0. To calculate the cutoff scores to maximize the ability to “rule in” DLD, we calculated the number and proportion of children in the DLD and TD groups whose scores were at or fell below (test positive) a given NRT (TOT PWC) value.

The number and proportion of children with a positive test result, and the LH ratios and prevalence for Total Words Correct for the Catalan–Spanish and European Portuguese-speaking children are shown in Table 6. As can be seen in Table 6, the LH ratio for a positive test result for the Catalan–Spanish-speaking children with DLD-CS compared to TD-CS controls for the Catalan version of the NRT was 33.00 (0.917/0.028), indicating that a child having a score of TOT-PWC of 70% or lower was 33 times more likely to be a child with DLD-CS as compared to a child with normal language. For the European Portuguese-speaking children with DLD-EP compared to TD-EP controls, the LH ratio for a positive test result was 35.00 (0.467/0.013), indicating that a TOT-PWC of 70% or lower was 35 times more likely to come from a child with DLD-EP as compared to one with normal language. These findings show that for the NRT-Cat and NRT-EPort, the best cut-point was the same, which was 70%. For the Spanish version of the NRT, the LH ratio for a positive test result was 33.88 (0.941/0.028), indicating that a TOT-PWC of 75% or lower was 33 times more likely to come from a child with DLD-CS as compared to a child with normal language.

#### 3.2.2. 2-Syllable Percentage of Words Correct (TOT PWC)

As can be seen in Table 7, the LH ratio for a positive test result for the Catalan–Spanish-speaking children with DLD-CS compared to TD-CS controls for the NRT-Cat was 22.00 (0.611/0.028), indicating that a TOT-PWC of 75% or lower for 2-syllable words was 22 times more likely to come from a child with DLD-CS as compared to a child with normal language. Similarly to NRT-Cat, the LH ratio for a positive test result for the European Portuguese-speaking children with DLD-EP compared to TD-EP controls for the NRT-EPort was 13.00 (0.520/0.040), indicating that a TOT-PWC of 75% or lower was 13 times more likely to come from a child with DLD-EP as compared to one with normal language. These findings show that for the NRT-Cat and NRT-EPort, the best cut-point was the same, which was 75%. For the NRT-Spa, the LH ratio for a positive test result was 7.41 (0.411/0.055), indicating that a TOT-PWC of 80% or lower for 2-syllable words was seven times more likely to come from a child with DLD-CS as compared to a child with normal language.

#### 3.2.3. 3-Syllable Percentage of Words Correct (TOT PWC)

As can be seen in Table 8, the LH ratio for a positive test result for the Catalan–Spanish-speaking children with DLD-CS compared to TD-CS controls for the NRT-Cat was 17.00 (0.472/0.028), indicating that a TOT-PWC of 62.5% or lower for 3-syllable words was 17 times more likely to come from a child with DLD-CS as compared to a child with normal language. Similarly to NRT-Cat, the LH ratio for a positive test result for the NRT-EPort for the European Portuguese-speaking children with DLD-EP compared to TD-EP controls was 22.00 (0.293/0.013), indicating that a TOT-PWC of 62.5% or lower was 22 times more likely to come from a child with DLD-EP as compared to a with normal language. These findings show that for the NRT-Cat and NRT-EPort, the best cut-point was the same, which was 62.5%. For the NRT-Spa, the LH ratio for a positive test result was 20.12 (0.559/0.028), indicating that a TOT-PWC of 70% or lower for 3- syllable words was 20 times more likely to come from a child with DLD-CS as compared to a child with normal language.

#### 3.2.4. 4-Syllable Percentage of Words Correct (TOT PWC)

As can be seen in Table 9, the LH ratio for a positive test result for the Catalan–Spanish-speaking children with DLD-CS compared to TD-CS controls for the NRT-Cat was 23.00 (0.639/0.028), indicating that a TOT-PWC of 62.5% or lower for 4-syllable words was 23 times more likely to come from a child with DLD-CS as compared to one with normal language. Similarly to NRT-Cat, the LH ratio for a positive test result for the NRT-EPort for the European Portuguese-speaking children with DLD-EP compared to TD-EP controls was 20.00 (0.267/0.013), indicating that a TOT-PWC of 62.5% or lower was 20 times more likely to come from a child with DLD-EP as compared to one with normal language. These findings show that for the NRT-Cat and NRT-EPort, the best cut-point was 62.5%. These findings show that for the NRT-Cat and NRT-EPort, the best cut-point was the same, which was 70%. For the NRT-Spa, the LH ratio for a positive test result was 27.53 (0.765/0.028), indicating that a TOT-PWC of 60% or lower for 4-syllable words was 27 times more likely to come from a child with DLD-CS as compared to a child with normal language.

#### 3.2.5. 5-Syllable Percentage of Words Correct (TOT PWC)

As can be seen in Table 10, the LH ratio for a positive test result for the Catalan–Spanish-speaking children with DLD-CS compared to TD-CS controls for the NRT-Cat was 29.00 (0.806/0.028), indicating that a TOT-PWC of 50% or lower for 5-syllable words was 29 times more likely to come from a child with DLD-CS as compared to a child with normal language. Similarly to NRT-Cat, the LH ratio for a positive test result for the NRT-EPort for the European Portuguese-speaking children with DLD-EP compared to TD-EP controls was 22.00 (0.293/0.013), indicating that a TOT-PWC of 62.5% or lower was 22 times more likely to come from a child with DLD-EP as compared to a child with normal language. Unlike the previous syllable length, the best cut-point differed. For the NRT-Spa, the LH ratio for a positive test result was 16.94 (0.941/0.056), indicating that a TOT-PWC of 55% or lower for 5-syllable words was 16 times more likely to come from a child with DLD-CS as compared to a child with normal language.

#### 3.2.6. 6-Syllable Percentage of Words Correct (TOT PWC)

As can be seen in Table 11, the LH ratio for a positive test result for the Catalan–Spanish-speaking children with DLD-CS compared to TD-CS controls for the NRT-Cat was 10.67 (0.889/0.083), indicating that a TOT-PWC of 25% or lower for 6-syllable words was 10 times more likely to come from a child with DLD-CS as compared to a child with normal language. In contrast, the LH ratio for a positive test result for the NRT-EPort for the *European Portuguese*-speaking children with DLD-EP compared to TD-EP controls was 18.33 (0.733/0.040), indicating that a TOT-PWC of 50% or lower was 18 times more likely to come from a child with DLD-EP as compared to a child with normal language. These findings show that for the NRT-Cat and NRT-EPort, the best cut-point differed.

#### 3.2.7. Summary of LH Ratios for Total Words Correct and by Syllable Length

There were similarities and differences in the cutoff points for total words percentage correct both overall and by syllable length for the two language cohorts and NRT. The absolute cutoff points having the best LH ratios for overall words produced correctly were similar for the NRT-Cat and NRT-EPort for ToT performance and for 2-, 3-, and 4-syllable lengths but differed for 5-syllable and 6-syllable lengths. However, the cut-points differed from the NRT-Spa. For the total performance for all three languages, a cut-point resulted in high LHs (i.e., greater than 20) based on Haynes et al. [77]. For 2-syllable length, NRT-Cat LHs were high and NRT-Span and NRT-EPort LHs were intermediate (i.e., 1–20). For 3-syllable lengths, NRT-Span and NRT-EPort LHs were high and that of NRT-Cat was intermediate but was close to the high cutoff (i.e., 17). For 4-syllable length, for all three languages, the LHs were all high. For 5-syllable length, NRT-Cat and NRT-EPort LHs were high and that of NRT-Span was intermediate but close to the high cutoff (i.e., 16.9). For 6-syllable length, the LHs were intermediate, with NRT-EPort LHs being close to high cutoff (i.e., 18.33).

### 3.3. Wordlikeness/Nonword Frequency

#### 3.3.1. High Wordlikeness/High Nonword Frequency (TOT PWC)

As can be seen in Table 12, for nonwords having high wordlikeness, the LH ratio for a positive test result for the Catalan–Spanish speaking children with DLD-CS compared to TD-CS controls for the NRT-Cat was 29.00 (0.806/0.028), indicating that a TOT-PWC of 65% or lower for nonwords having high wordlikeness regardless of syllable length was 29 times more likely to come from a child with DLD-CS as compared to a child having normal language. For nonwords having high wordlikeness, the LH ratio for a positive test result for the NRT-EPort for the European Portuguese-speaking children with DLD-EP compared to TD-EP controls was 39.00 (0.520/0.013), indicating that a TOT-PWC of 75% or lower was 39 times more likely to come from a child with DLD-EP as compared to a child having normal language. These findings show that for the NRT-Cat and NRT-EPort, the best cut-point differed. For the Spanish NRT for high-frequency nonwords, the LH ratio for a positive test result was 16.41 (0.912/0.056), indicating that a TOT-PWC of 80% or lower for nonwords having high nonword frequency regardless of syllable length was 16 times more likely to come from a child with DLD-CS as compared to a child with normal language.

#### 3.3.2. Low Wordlikeness/Low Nonword Frequency (TOT PWC)

As can be seen in Table 13, for nonwords having low wordlikeness, the LH ratio for a positive test result for the Catalan–Spanish-speaking children with DLD-CS compared to TD-CS controls for the NRT-Cat was 31.00 (0.861/0.028), indicating that a TOT-PWC of 65% or lower for nonwords having low wordlikeness regardless of syllable length was 31 times more likely to come from a child with DLD-CS as compared to a child with normal language. In contrast, for nonwords having low wordlikeness, the LH ratio for a positive test result for the NRT-EPort for the European Portuguese children with DLD-EP compared to TD-EP controls was 35.00 (0.467/0.013), indicating that a TOT-PWC of 65% or lower was 35 times more likely to come from a child with DLD-EP as compared to a child with normal language. These findings show that for the NRT-Cat and NRT-EPort, the best cut-point was the same, which was 65%. For the NRT-Spa, the LH ratio for a positive test result was 14.82 (0.824/0.056), indicating that a TOT-PWC of 65% or lower for nonwords having low nonword frequency regardless of syllable length was 14 times more likely to come from a child with DLD-CS as compared to a child with normal language.

#### 3.3.3. Summary of LH Ratios for Wordlikeness/Nonword Frequency

For nonwords having high wordlikeness ratings, although the absolute cut-points differed for the Catalan and European Portuguese NRTs, the LH for both cohorts fell in the high range, according to Haynes et al. [77]. For the Spanish NRT, the LH for high nonword frequency was in the intermediate range. For nonwords having low wordlikeness for the Catalan and European Portuguese, the NRTs were the same and the LH for both cohorts again fell in the high range according to Haynes et al. [77]. For low-frequency nonwords, although the absolute cut-points were the *same* as those of the NRT-Cat and NRT-EPort, for the NRT-Spa, the LH fell in the intermediate high range according to Haynes et al. [77]. Taken together, the findings from the wordlikeness versus nonword frequency comparison indicates that wordlikeness may be more sensitive to DLD diagnosis regardless of the language used as compared to nonword frequency with the LHs for wordlikeness being more than double those of word frequency.

## 4. Summary of Results and Discussion

In this study, we investigated the diagnostic accuracy of using a nonword repetition task to discriminate DLD for TD across three different languages. For two of the three languages (Catalan and European Portuguese), NRT lists were created and administered using the same method, and for Spanish NRT, we used an existing list from Aguado [64]. Although there were absolute accuracy differences across the Catalan–Spanish and European Portuguese-speaking children, a similar pattern of performance accuracy was observed for both syllable length and wordlikeness (NRT-Cat and NRT-Port)/nonword frequency (NRT-Spa) across the two cohorts. Specifically, the children with DLD performed worse than their TD peers across all syllable lengths, with performance worsening as the syllable length increased.

For the LH analysis, a strikingly similar pattern was observed across the two cohorts in the pattern of cut-points for the LH ratios both for overall performance, syllable length, wordlikeness, and nonword frequency. Importantly, the similarities were highest for the Catalan and European Portuguese NRT. Specifically, the LHs for the two versions mirror each other both for syllable length and wordlikeness, where very high LH values were observed for both high and low wordlikness. This pattern was not evident in the Spanish NRT based on the frequency of the nonwords.

Children in both cohort groups with DLD repeated nonwords with high wordlikeness with greater accuracy than those with low wordlikeness, which is a pattern that was not evident in the TD controls. This finding was in line with past research, which has found similar patterns with children with DLD performing better with high wordlikeness and/or high phonotactic frequency nonwords, whereas this performance difference is not as extreme in the TD controls [25,78]. As has been mentioned previously, nonword repetition ability correlates with language proficiency, but it also correlates with the amount of language exposure in both typical and clinical populations [43]. With greater language exposure and proficiency comes greater long-term linguistic representations. This means that although a nonword may have low phonotactic probability or low wordlikeness, a child with larger exposure and proficiency has been exposed to more instances of words that conform to the low phonotactic/wordlikeness nonwords, and as such, they are more common for these children. On the other hand, children with DLD have limited linguistic representations within their extant long-term memory, and as such, the difference between low wordlikeness/frequency nonwords and high wordlikeness/frequency nonwords is much starker than for their TD peers, resulting in an advantageous interaction for high wordlikeness/frequency that is not found in the TD populations.

In this study, although performance for both groups of children with DLD was lower than that of their respective TD peers, overall, the bilingual Catalan–Spanish-speaking children had worse performance than the monolingual children who speak European Portuguese. This finding adds to previous literature showing that bilingual children perform less accurately during NRT than their monolingual peers [43,79]. These previous studies investigated sequentially bilingual children and found that these children were less accurate when repeating nonwords in the language with which they had less exposure and less proficiency than their monolingual peers. An additional study compared the NRT accuracy in four groups of children (monolingual English TD and DLD, and bilingual Spanish–English TD and DLD), and it found that the bilingual Spanish–English children performed worse on English nonwords than the respective monolingual groups, but the bilingual children with DLD outperformed the monolingual English DLD group on Spanish nonwords, with all three groups outperformed by the bilingual TD children [34].

It is important for the purposes of the current study that despite the overall difference in performance accuracy between the two language groups (Catalan–Spanish, European Portuguese) and language status (bilingual vs. monolingual), the LH values remained constant and nearly identical across the groups. In the case of the Catalan and European Portuguese languages, a possible reason for this stability may be the identical method used to create the nonword lists for both language cohorts. However, although the LH values found for the Spanish NRT lists were at times lower, they were also highly consistent with the other language lists. This consistency in LH values strongly suggests the validity of utilizing NRT to distinguish DLD from TD across a range of linguistically diverse populations, even when similar methods are not used to create NRT lists.

Finally, the high LH values that we found across our four groups may be a result of (1) the unpredictable presentation order of the nonwords for the Catalan and European Portugese NRT; and (2) differences in the wordlikeness ratings of the nonwords in the NRT-Cat and NRT-EPort stimuli. Specifically, in our study, the Catalan and European Portuguese nonwords were not presented sequentially in accordance to syllable length (i.e., all 2-syllable nonwords followed by all 3-syllable nonwords, etc.). Instead, the nonwords in our lists were presented in a fixed random order, such that participants could not predict the syllable length or wordlikeness of an upcoming nonword. The unpredictability of stimuli presentation has been found to impact task difficulty and subsequent performance in studies of adult cognition [80] and may have increased the difficulty in the NRT that effectively negated the ceiling effect for our four groups. However, it is worth noting that few studies have reported whether the presentation of their nonwords were sequential or randomized, and as such, this interpretation of results must remain somewhat speculative. Future research should investigate the influence of the order of nonword stimuli presentation on accuracy to ensure that behavioral results are truly representative of the cognitive processes that underlie nonword repetition itself, and not an artifact of stimuli presentation methodology.

Taken together, the findings from this study suggest that in the absence of pre-existing standardized NRT, to use NRT as a potential identifier of DLD, an NRT task can be developed from scratch in a child’s native language using the method outlined in this study for the NRT-Cat and NRT-EPort. Furthermore, the findings from this study show that using 3-, 4-, and 5-syllable nonwords that are presented in a random order is a valuable presentation approach. Finally, using nonwords that vary in wordlikeness may result in the most sensitive version of an NRT task. Surprisingly, and encouragingly, for languages where large-scale word databases are not available to calculate measures such as word frequency, phonotactic probability, etc., the findings from this study show that using a native speaker’s ranking of wordlikeness of nonwords results in a valuable measure that appears to increase the diagnostic sensitivity of an NRT task.

There has been a growing interest in the development of behavioral markers of DLD. For the majority of children with DLD, the deficits extend beyond the language system to include deficits in the nonverbal and cognitive domains as well. If unidentified or left untreated, these deficits persist into adulthood, putting these children at risk for failure in academic and work settings, lower standard of living, social isolation, and significant secondary socio-emotional and stress-related health issues. The variability in the DLD language deficit profile coupled with a diagnostic classification system based on exclusion criteria makes the identification and effective treatment of DLD a significant challenge to both researchers and clinicians. The identification of markers for the objective diagnosis of DLD have broad health-related implications. Specifically, tasks that are not language-specific and that have the ability to differentiate DLD from typically developing children have the potential to aid in the refinement of the diagnostic accuracy of DLD, help identify potential subtypes of the disorder, resulting in better estimations of prognosis for the disorder and predictive validity of individual symptoms of DLD, and aid in the development of more effective therapeutic approaches that are more tightly linked to the underlying cause(s) of the disorder.

## Figures and Tables

**Table 1:** Age and standardized scores for language and cognitive assessment measures for Catalan–Spanish children with developmental language disorder (DLD-CS) and typically developing (TD-CS) children.

	DLD-CS (*n* = 36)			TD-CS (*n* = 36)			Comparison	
Variable	Mean	SD	Range	Mean	SD	Range	*t*(70)	*p*
Age in months	107.72	28.76	64–185	111.58	28.68	67–194	−0.57	*p* = 0.57
K-BIT mat (NVIQ) ^a^	98.67	12.10	76–119	102.53	8.82	88–124	−1.54	*p =* 0.13
CELF-CLS ^b^	73.78	10.98	45–89	109.83	6.57	99–130	−16.91	*p* < 0.01
CELF-ELS ^c^	73.86	8.69	52–87	109.39	7.73	89–128	−18.33	*p* < 0.01
CELF-RLS ^d^	78.92	9.74	59–97	105.44	5.89	93–118	−13.98	*p* < 0.01

Note. For each variable, age-scaled scores have a mean of 100 and an SD of 15 (except age in months). ^a^ K-BIT mat = Kaufman Brief Intelligence; Matrices subtest, Spanish version [58]. NVIQ: Non-verbal intelligence. ^b^ CELF-4 CLS = Spanish Clinical Evaluation of Language Fundamentals, 4th edition: Core Language score [59]. ^c^ CELF-4 ELS = Spanish Clinical Evaluation of Language Fundamentals, Fourth Edition: Expressive Language score [59]. ^d^ CELF-4 RLS = Spanish Clinical Evaluation of Language Fundamentals, Fourth Edition: Receptive Language score [59].

**Table 2:** The nonwords for the Catalan version of the nonword repetition task (NRT-Cat) by syllable length and wordlikeness.

2-Syllable	3-Syllable	4-Syllable	5-Syllable	6-Syllable
High Wordlikeness Nonwords				
marlut	grimpola	palimatrenc	balatenesta	pomalimenasa
calim	llendira	xipileta	jolistarista	desperdiculandre
padot	vifatull	calisota	xepunetura	parmicaginosa
galmet	dubalet	clomitura	parantalaresc	situcalinomi
Low Wordlikeness Nonwords				
pidop	satompa	becuradoc	lamerquitorma	diraculmestici
rolma	xolopi	daltrosqueti	lifortamasuc	purmesidocata
becra	calmepi	castretuma	lemulticada	casitedilafa
dapa	dalumi	milusota	zicuparamal	garelisupota

**Table 3:** The nonwords for the European Portuguese version of the nonword repetition task (NRT-EPort) by syllable length and wordlikeness.

2-Syllable	3-Syllable	4-Syllable	5-Syllable	6-Syllable
High Wordlikeness Nonwords				
naca	lofena	covilado	melanifito	turamisalato
fopa	banita	fenerade	bonifadade	detagapalico
trana	praleta	trapilado	craletonina	prinalvenioso
prota	bramato	cravastado	versatranista	volturacidade
Low Wordlikeness Nonwords				
cafo	mafopa	lemanado	nocafozano	rolinicistato
tuma	dopeta	dilomopa	lodanapito	fatatuviricho
grapa	gremata	dragamato	defermicato	satopogatico
trila	tramafa	trafeleste	promoflicada	cremoforosada

**Table 4:** Means and standard deviations on the NRT task for percentage of words correct (PWC) overall and for each syllable length for the Catalan and Spanish versions of the NRT for the Catalan–Spanish (CS) and European Portuguese (EP) children with developmental language disorder (DLD) and typically developing (TD) controls.

		Nonword Length				
Group	2-syll PWC	3-syll PWC	4-syll PWC	5-syll PWC	6-syll PWC	Total PWC
	**CS-Catalan**					
DLD ^a^ (*n* = 36)	70.8 (21.7)	68.4 (21.2)	56.9 (23.9)	31.5 (25.6)	12.1 (18.0)	48.0 (18.0)
TD ^b^ (*n* = 36)	97.9 (5.5)	93.4 (10.1)	92.0 (10.4)	84.0 (12.1)	73.6 (24.9)	88.0 (8.0)
	**CS-Spanish**					
DLD ^a^ (*n* = 34)	82.2 (14.3)	68.5 (21.7)	45.0 (21.8)	25.2 (21.3)	-	54.5 (16.9)
TD ^b^ (*n* = 36)	95.2 (5.5)	92.3 (7.5)	86.6 (11.2)	80.9 (15.2)	-	89.0 (7.3)
	**European Portuguese**					
DLD ^a^ (*n* = 75)	77.1 (15.5)	75.5 (13.5)	76.6 (16.2)	77.1 (20.7)	48.8 (17.2)	71.0 (12.2)
TD ^b^ (*n* = 75)	95.6 (6.9)	92.6 (9.2)	96.3 (7.3)	95.3 (7.6)	87.0 (13.2)	93.4 (5.5)

^a^ Developmental Language Disorder. ^b^ Typically Developing. Note. The Spanish NRT version does not include nonwords of 6-syllable length.

**Table 5:** Means and standard deviations on the NRT for percentage of words correct (PWC) for high and low wordlikeness for the Catalan and Spanish versions of the NRT for the Catalan–Spanish (CS) and European Portuguese (EP) children with developmental language disorder (DLD) and typically developing (TD) controls.

Group	High Wordlikeness	Low Wordlikeness
**CS-Catalan**		
DLD ^a^ (*n* = 36)	52.0 (18.8)	44.0 (19.0)
TD ^b^ (*n* = 36)	87.6 (9.3)	88.6 (9.4)
**CS-Spanish**		
DLD ^a^ (*n* = 34)	62.3 (17.2)	47.9 (19.5)
TD ^b^ (*n* = 36)	92.6 (6.5)	85.4 (10.2)
**European Portuguese**		
DLD ^a^ (*n* = 75)	75.1 (11.9)	67.0 (15.1)
TD ^b^ (*n* = 75)	94.8 (5.4)	91.9 (7.6)

^a^ Developmental Language Disorder. ^b^ Typically Developing.

**Table 6:** The number and proportion of children with a positive test result (ruling in disorder), Prevalence and Likelihood Ratio (LH) for each of the cutoff values based on total word percent correct (TOT-PWC) on the NRT for the Catalan–Spanish (CS) and European Portuguese (EP) children with developmental language disorder (DLD) and typically developing (TD) controls.

TOT-PWC	DLD (True Positive)		TD (False Positive)		Prevalence	LH
Cutoff	No.	Prop.	No.	Prop.		
**CS-Catalan**						
≤65.0	30	0.833	1	0.028	0.50	30.00
**≤70.0**	**33**	**0.917**	**1**	**0.028**	**0.50**	**33.00**
≤75.0	34	0.944	3	0.083	0.50	11.33
≤80.0	35	0.972	7	0.194	0.50	5.00
≤85.0	35	0.972	10	0.278	0.50	3.50
≤90.0	36	1.00	21	0.583	0.50	1.71
≤95.0	36	1.00	32	0.889	0.50	1.13
≤100	36	1.00	36	1.00	0.50	1.00
**CS-Spanish**						
≤65.0	25	0.735	0	0.000	0.48	Inf.
≤70.0	27	0.794	1	0.028	0.48	28.58
**≤75.0**	**32**	**0.941**	**1**	**0.028**	0.48	**33.88**
≤80.0	32	0.941	6	0.166	0.48	5.65
≤85.0	34	1.00	12	0.333	0.48	3.00
≤90.0	34	1.00	18	0.500	0.48	2.00
≤95.0	34	1.00	29	0.805	0.48	1.24
≤100	34	1.00	36	1.00	0.48	1.00
**European Portuguese**						
≤65.0	22	0.293	0	0.000	0.50	Inf.
**≤70.0**	**35**	**0.467**	**1**	**0.013**	**0.50**	**35.00**
≤75.0	46	0.613	2	0.027	0.50	23.00
≤80.0	59	0.787	2	0.027	0.50	29.50
≤85.0	72	0.960	7	0.093	0.50	10.29
≤90.0	74	0.987	18	0.240	0.50	4.11
≤95.0	75	1.00	51	0.680	0.50	1.47
≤100	75	1.00	75	1.00	0.50	1.00

Note. Values ≤ 65.0 cutoffs are not presented because LH = Inf. The cutoffs for best LH are highlighted in bold.

**Table 7:** The number and proportion of children with a positive test result (ruling in disorder), prevalence and likelihood ratio (LH) for each of the cutoff value based on total word percentage correct (TOT-PWC) for 2-syllable length nonwords for the Catalan–Spanish (CS) and European Portuguese (EP) children with developmental language disorder (DLD) and typically developing (TD) controls.

2-syll TOT-PWC	DLD (True Positive)		TD (False Positive)		Prevalence	LH
Cutoff	No.	Prop.	No.	Prop.		
**CS-Catalan**						
**≤75.0**	**22**	**0.611**	**1**	**0.028**	**0.50**	**22.00**
≤87.5	31	0.861	6	0.167	0.50	5.16
≤100	36	1.00	36	1.00	0.50	1.00
**CS-Spanish**						
≤75.0	8	0.235	0	0.000	0.48	Inf.
**≤80.0**	**14**	**0.411**	**2**	**0.055**	0.48	**7.41**
≤85.0	17	0.500	3	0.083	0.48	6.00
≤90.0	27	0.794	9	0.250	0.48	3.18
≤95.0	32	0.941	20	0.555	0.48	1.69
≤100	34	1.00	36	1.00	0.48	1.00
**European Portuguese**						
**≤75.0**	**39**	**0.520**	**3**	**0.040**	**0.50**	**13.00**
≤87.5	67	0.893	23	0.307	0.50	2.91
≤100	75	1.00	75	1.00	0.50	1.00

Note. The possible cutoffs for Spanish NRT are different from those for Catalan and European Portuguese NRT because for the Spanish version of the task, there are 20 nonwords for each syllable length and for the Catalan and European Portuguese versions, there are eight nonwords for each syllable length. Values ≤ 75.0 cutoffs are not presented because LH = Inf. The cutoffs for best LH are highlighted in bold.

**Table 8:** The number and proportion of children with a positive test result (ruling in disorder), prevalence and likelihood ratio (LH) for each of the cutoff value based on total word percentage correct (TOT-PWC) for 3-syllable nonwords for the Catalan–Spanish (CS) and European Portuguese (EP) children with developmental language disorder (DLD) and typically developing (TD) controls.

3-syll TOT-PWC	DLD (True Positive)		TD (False Positive)		Prevalence	LH
Cutoff	No.	Prop.	No.	Prop.		
**CS-Catalan**						
≤50.0	5	0.139	1	0.028	0.50	5.00
**≤62.5**	**17**	**0.472**	**1**	**0.028**	**0.50**	**17.00**
<75.0	25	0.694	2	0.056	0.50	12.50
≤87.5	35	0.972	16	0.444	0.50	2.19
≤100	36	1.00	36	1.00	0.50	1.00
**CS-Spanish**						
≤55.0	9	0.264	0	0.000	0.48	Inf.
≤60.0	11	0.324	0	0.000	0.48	Inf.
≤65.0	12	0.353	0	0.000	0.48	Inf.
**≤70.0**	**19**	**0.559**	**1**	**0.028**	0.48	**20.12**
≤75.0	21	0.618	2	0.056	0.48	11.12
≤80.0	23	0.676	3	0.083	0.48	8.12
≤85.0	27	0.794	8	0.222	0.48	3.57
≤90.0	32	0.941	16	0.444	0.48	2.12
≤95.0	33	0.971	25	0.694	0.48	1.40
≤100	34	1.00	36	1.00	0.48	1.00
**European Portuguese**						
≤50.0	5	0.067	0	0.000	0.50	Inf.
**≤62.5**	**22**	**0.293**	**1**	**0.013**	**0.50**	**22.00**
<75.0	47	0.627	9	0.120	0.50	5.22
≤87.5	71	0.947	34	0.453	0.50	2.09
≤100	75	1.00	75	1.00	0.50	1.00

Note. The possible cut-points for Spanish NRT are different from Catalan and European Portuguese NRT because for the Spanish version of the task, there are 20 nonwords for each syllable length, and for Catalan and European Portuguese, there are eight nonwords for each syllable length. Values ≤ 50.0/55.0 cutoffs are not presented, because LH = Inf. The cutoffs for best LH are highlighted in bold.

**Table 9:** The number and proportion of children with a positive test result (ruling in disorder), prevalence and likelihood ratio (LH) for each of the cutoff values based on total word percentage correct (TOT-PWC) for 4-syllable length nonwords for the Catalan–Spanish (CS) and European Portuguese (EP) children with developmental language disorder (DLD) and typically developing (TD) controls.

4-syll TOT-PWC	DLD (True Positive)		TD (False Positive)		Prevalence	LH
Cutoff	No.	Prop.	No.	Prop.		
**CS-Catalan**						
≤50.0	17	0.472	0	0.028	0.50	Inf.
**≤62.5**	**23**	**0.639**	**1**	**0.028**	**0.50**	**23.00**
<75.0	31	0.861	6	0.017	0.50	5.17
≤87.5	34	0.944	16	0.444	0.50	2.13
≤100	36	1.00	36	1.00	0.50	1.00
**CS-Spanish**						
≤55.0	23	0.676	1	0.028	0.48	24.35
**≤60.0**	**26**	**0.765**	**1**	**0.028**	**0.48**	**27.53**
≤65.0	27	0.794	3	0.083	0.48	9.53
≤70.0	30	0.882	4	0.111	0.48	7.94
≤75.0	33	0.971	7	0.194	0.48	4.99
≤80.0	34	1.00	11	0.306	0.48	3.27
≤85.0	34	1.00	15	0.417	0.48	2.40
≤90.0	34	1.00	25	0.694	0.48	1.44
≤95.0	34	1.00	29	0.806	0.48	1.24
≤100	34	1.00	36	1.00	0.48	1.00
**European Portuguese**						
≤50.0	8	0.107	0	0.000	0.50	Inf.
**≤62.5**	**20**	**0.267**	**1**	**0.013**	**0.50**	**20.00**
<75.0	46	0.613	3	0.040	0.50	15.33
≤87.5	63	0.840	18	0.240	0.50	3.50
≤100	75	1.00	75	1.00	0.50	1.00

Note. The possible cut-points for Spanish NRT are different from Catalan and European Portuguese NRT, because for the Spanish version of the task, there are 20 nonwords for each syllable length, and for Catalan and European Portuguese, there are eight nonwords for each syllable length. Values ≤ 50.0/55.0 cutoffs are not presented, because LH = Inf. The cutoffs for the best LH are highlighted in bold.

**Table 10:** The number and proportion of children with a positive test result (ruling in disorder), prevalence and likelihood ratio (LH) for each of the cutoff value based on total word percentage correct (TOT-PWC) for 5-syllable length nonwords for the Catalan–Spanish (CS) and European Portuguese (EP) children with developmental language disorder (DLD) and typically developing (TD) controls.

5-syll TOT-PWC	DLD (True Positive)		TD (False Positive)		Prevalence	LH
Cutoff	No.	Prop.	No.	Prop.		
**CS-Catalan**						
**≤50.0**	**29**	**0.806**	**1**	**0.028**	**0.50**	**29.00**
≤62.5	31	0.861	5	0.139	0.50	6.20
<75.0	35	0.972	10	0.278	0.50	3.50
≤87.5	35	0.972	29	0.805	0.50	1.20
≤100	36	1.000	36	1.000	0.50	1.00
**CS-Spanish**						
≤45.0	27	0.794	2	0.056	0.48	14.29
**≤55.0**	**32**	**0.941**	**2**	**0.056**	0.48	**16.94**
≤60.0	33	0.971	5	0.139	0.48	6.99
≤65.0	33	0.971	7	0.194	0.48	4.99
≤70.0	33	0.971	9	0.250	0.48	3.88
≤75.0	33	0.971	12	0.333	0.48	2.91
≤80.0	34	1.00	15	0.417	0.48	2.40
≤85.0	34	1.00	24	0.667	0.48	1.50
≤90.0	34	1.00	26	0.722	0.48	1.38
≤95.0	34	1.00	32	0.889	0.48	1.13
≤100	34	1.00	36	1.00	0.48	1.00
**European Portuguese**						
≤50.0	14	0.187	0	0.000	0.50	Inf.
**≤62.5**	**22**	**0.293**	**1**	**0.013**	**0.50**	**22.00**
<75.0	35	0.467	3	0.040	0.50	11.67
≤87.5	58	0.773	24	.320	0.50	2.42
≤100	75	1.00	75	1.00	0.50	1.00

Note. The possible cut-points for Spanish NRT are different from Catalan and European Portuguese NRT because for the Spanish version of the task, there are 20 nonwords for each syllable length, and for Catalan and European Portuguese, there are eight nonwords for each syllable length. Values ≤ 45.0/50.0 cutoffs are not presented, because LH = Inf. The cutoffs for the best LH are highlighted in bold.

**Table 11:** The number and proportion of children with a positive test result (ruling in disorder), prevalence and likelihood ratio (LH) for each of the cutoff value based on total word percentage correct (TOT-PWC) for 6-syllable length nonwords for the Catalan–Spanish (CS) and European Portuguese (EP) children with developmental language disorder (DLD) and typically developing (TD) controls.

6-syll TOT-PWC	DLD (True Positive)		TD (False Positive)		Prevalence	LH
Cutoff	No.	Prop.	No.	Prop.		
**CS-Catalan**						
**≤25.0**	**32**	**0.889**	**3**	**0.083**	**0.50**	**10.67**
≤37.5	33	0.917	4	0.111	0.50	8.25
≤50.0	34	0.944	6	0.167	0.50	5.67
≤62.5	35	0.972	15	0.417	0.50	2.33
<75.0	36	1.00	20	0.556	0.50	1.80
≤87.5	36	1.00	26	0.722	0.50	1.38
≤100	36	1.00	36	1.00	0.50	1.00
**European Portuguese**						
≤25.0	8	0.107	0	0.000	0.50	Inf.
≤37.5	30	0.400	0	0.000	0.50	Inf.
**≤50.0**	**55**	**0.733**	**3**	**0.040**	**0.50**	**18.33**
≤62.5	64	0.853	8	0.107	0.50	8.00
<75.0	73	0.973	18	0.240	0.50	4.06
≤87.5	74	0.987	49	0.653	0.50	1.51
≤100	75	1.00	75	1.00	0.50	1.00

Note. The Spanish NRT version does not include nonwords of 6-syllable length. Values ≤ 25.0 cutoffs are not presented, because LH = Inf. The cutoffs for best LH are highlighted in bold.

**Table 12:** The number and proportion of children with a positive test result (ruling in disorder), prevalence, and likelihood ratio (LH) for each of the cutoff values based on total word percentage correct (TOT-PWC) for HIGH wordlikeness (HWL) nonwords for Catalan and Portuguese NRT and HIGH nonword frequency (HNF) nonwords for Spanish NRT for the Catalan–Spanish (CS) and European Portuguese (EP) children with developmental language disorder (DLD) and typically developing (TD) controls.

HWL/NF TOT-PWC	DLD (True Positive)		TD (False Positive)		Prevalence	LH
Cutoff	No.	Prop.	No.	Prop.		
**CS-Catalan**						
≤60.0	26	0.722	1	0.028	0.50	26.00
**≤65.0**	**29**	**0.806**	**1**	**0.028**	**0.50**	**29.00**
≤70.0	31	0.861	3	0.083	0.50	10.33
≤75.0	33	0.917	5	0.139	0.50	6.60
≤80.0	34	0.944	9	0.250	0.50	3.78
≤85.0	35	0.972	14	0.389	0.50	2.50
≤90.0	36	1.00	24	0.667	0.50	1.50
≤95.0	36	1.00	32	0.889	0.50	1.13
≤100	36	1.00	36	1.00	0.50	1.00
**CS-Spanish**						
≤60.0	14	0.412	0	0.000	0.48	Inf.
≤65.0	17	0.500	0	0.000	0.48	Inf.
≤70.0	22	0.647	0	0.000	0.48	Inf.
≤75.0	27	0.794	2	0.056	0.48	14.29
**≤80.0**	**31**	**0.912**	**2**	**0.056**	0.48	**16.41**
≤85.0	33	0.971	3	0.083	0.48	11.65
≤90.0	34	1.00	10	0.278	0.48	3.60
≤95.0	34	1.00	26	0.722	0.48	1.38
≤100	34	1.00	36	1.00	0.48	1.00
**European Portuguese**						
≤60.0	11	0.147	0	0.000	0.50	Inf.
≤65.0	17	0.227	0	0.000	0.50	Inf.
≤70.0	22	0.293	0	0.000	0.50	Inf.
**≤75.0**	**39**	**0.520**	**1**	**0.013**	**0.50**	**39.00**
≤80.0	56	0.747	3	0.040	0.50	18.67
≤85.0	67	0.893	7	0.093	0.50	9.57
≤90.0	72	0.960	18	0.240	0.50	4.00
≤95.0	75	1.00	48	0.640	0.50	1.56
≤100	75	.227	75	1.00	0.50	1.00

Note. Values ≤ 60.0 cutoffs are not presented because LH = Inf. The cutoffs for best LH are highlighted in bold.

**Table 13:** The number and proportion of children with a positive test result (ruling in disorder), prevalence and likelihood ratio (LH) for each of the cutoff value based on total word percentage correct (TOT-PWC) for LOW wordlikeness (LWL) nonwords for Catalan and Portuguese NRT (NRT-Cat and NRT-Port) and LOW nonword frequency (HNF) nonwords for Spanish NRT (NRT-Spa) for the Catalan–Spanish (CS) and European Portuguese (EP) children with developmental language disorder (DLD) and typically developing (TD) controls.

LWL/LNF TOT-PWC	DLD (True Positive)		TD (False Positive)		Prevalence	LH
Cutoff	No.	Prop.	No.	Prop.		
**CS-Catalan**						
≤55.0	28	0.778	1	0.028	0.50	28.00
≤60.0	29	0.806	1	0.028	0.50	29.00
**≤65.0**	**31**	**0.861**	**1**	**0.028**	**0.50**	**31.00**
≤70.0	34	0.944	2	0.056	0.50	17.00
≤75.0	34	0.944	4	0.111	0.50	8.50
≤80.0	35	0.972	7	0.194	0.50	5.00
≤85.0	36	1.00	14	0.389	0.50	2.57
≤90.0	36	1.00	19	0.528	0.50	1.89
≤95.0	36	1.00	33	0.917	0.50	1.09
≤100	36	1.00	36	1.00	0.50	1.00
**CS-Spanish**						
≤55.0	21	0.618	0	0.000	0.48	Inf.
≤60.0	24	0.706	0	0.000	0.48	Inf.
**≤65.0**	**28**	**0.824**	**2**	**0.056**	0.48	**14.82**
≤70.0	30	0.882	6	0.166	0.48	5.29
≤75.0	32	0.941	7	0.194	0.48	4.84
≤80.0	33	0.971	10	0.278	0.48	3.49
≤85.0	34	1.00	17	0.472	0.48	2.12
≤90.0	34	1.00	24	0.667	0.48	1.50
≤95.0	34	1.00	30	0.833	0.48	1.20
≤100	34	1.00	36	1.00	0.48	1.00
**European Portuguese**						
≤55.0	20	0.267	1	0.013	0.50	20.00
≤60.0	27	0.360	1	0.013	0.50	27.00
**≤65.0**	**35**	**0.467**	**1**	**0.013**	**0.50**	**35.00**
≤70.0	42	0.560	2	0.027	0.50	21.00
≤75.0	54	0.720	4	0.053	0.50	13.50
≤80.0	64	0.853	6	0.080	0.50	10.67
≤85.0	72	0.960	14	0.187	0.50	5.14
≤90.0	73	0.973	31	0.413	0.50	2.35
≤95.0	75	1.00	61	0.813	0.50	1.23
≤100	75	1.00	75	1.00	0.50	1.00

Note. Values ≤ 55.0 cutoffs are not presented because LH = Inf. The cutoffs for best LH are highlighted in bold.

## Data Availability

The data that support the findings of this study are available from the corresponding author upon reasonable request.

## Appendix A

**Table A1:** The phonetic transcriptions in International Phonetic Transcription (IPA) of the nonwords for the Catalan version of the Nonword Repetition task (NRT-Cat) by syllable length and wordlikeness.

2-Syllable	3-Syllable	4-Syllable	5-Syllable	6-Syllable
High wordlikeness nonwords				
Marlut [mɐr. ˈlut]	Grimpola [ɡɾim.ˈpa.lə]	Palimatrenc [pɐ.li.mɐ.ˈtɾɛɳk]	Balatenesta [bɐ.lɐ.tə.ˈnɛs.ta]	Pomalimenasa [pʊ.mɐ.li.mɐ.ˈna.zɐ]
Calim [ka.ˈlim]	Llendira [ʎən.ˈdi.ɾɐ]	Xipileta [ɕi.pi.ˈlɛ.tɐ]	Jolistarista [ʑʊ.lis.tɐ.ˈɾis.tə]	Desperdiculandre [dəs.pəɾ.ði.ku.ˈlan.dɾə]
Padot [pɐ.ˈðɔt]	Vifatull [vi.fa.ˈtuʎ]	Calisota [kɐ.li.ˈsɔ.tə]	Xepunetura [ɕə.pʊ.nə.ˈtu.ɾɐ]	Parmicaginosa [pɐr.mi.kɐ.ʑi.ˈno.zə]
Galmet [gɐl.ˈmɛt]	Dubalet [du.βɐ.ˈlɛt]	Clomitura [klu.mi.ˈtu. ɾa]	Parantalaresc [pa.ɾən.tɐ.lɐ.ˈɾɛsk]	Situcalinomi [si.tʊ.kɐ.li.ˈnɔ.mi]
low Wordlikeness nonwords				
Pidop [pi.ˈðɔp]	Satompa [sɐ.ˈtum.pa]	Becuradoc [bɐ.kʊ.ɾɐ.ˈðɔk]	Lamerquitorma [la.mar.ki.ˈtor.mɐ]	Diraculmestici [di.ɾɐ.kʊl.mas.ˈti.si]
Rolma [ˈrol.ma]	Xolopi [ɕʊ.ˈlɔ.pi]	Daltrosqueti [dɐl.tɾʊs.ˈkɛ.ti]	Lifortamasuc [li.for.tɐ.mɐ.ˈsuk]	Purmesidocata [pur.mə.si.ðʊ.ˈca.tə]
Becra [ˈbɛ.kɾɐ]	Calmepi [kɐl.ˈmɛ.pi]	Castretuma [kɐs.tɾɐ.ˈtu.mə]	Lemulticada [lɐ.mul.ti.ˈka.ðə]	Casitedilafa [ka.si.ta.di.ˈla.fɐ]
Dapa [ˈðæ.pa]	Dalumi [ða.ˈlu.mi]	Milusota [mi.lʊ.ˈsɔ.tɐ]	Zicuparamal [zi.ku.pɐ.ɾɐ.ˈmal]	Garelisupota [gɐ.ɾɐ.li.su.ˈpɔ.tə]

## Appendix B

**Table A2:** The phonetic transcriptions in International Phonetic Transcription (IPA) of the nonwords for the European Portuguese version of the Nonword Repetition Task (NRT-EPort) by syllable length and wordlikeness.

2-Syllable	3-Syllable	4-Syllable	5-Syllable	6-Syllable
High wordlikeness nonwords				
Naca [ˈna.kɐ]	Lofena [lu.ˈfe.nɐ]	Covilado [ku.vi.ˈla.du]	Melanifito [mɨ.lɐ.nɨ.ˈfi.tu]	Turamisalato [tu.rɐ.mi.zɐ.ˈla.tu]
Fopa [ˈfo.pɐ]	Banita [bɐ.ˈni.tɐ]	Fenerade [fɨ.nɨ.ˈɾa.dɨ]	Bonifadade [bɔ.nɨ.fɐ.ˈda.dɨ]	Detagapalico [dɨ.tɐ.gɐ.pɐ.ˈli.ku]
Trana [ˈtrɐ.nɐ]	Praleta [pra.ˈle.tɐ]	Trapilado [trɐ.pi.ˈla.du]	Craletonina [krɐ.lɨ.tu.ˈni.nɐ]	Prinalvenioso [pri.ɐɫ.vɨ.ˈnjo.zu]
Prota [ˈprɔ.tɐ]	Bramato [brɐ.ˈma.tu]	Cravastado [krɐ.vɐʃ.ˈta.du]	Versatranista [vεɾ.sɐ.trɐ.ˈniʃ.tɐ]	Volturacidade [vɔɫ.tu.rɐ.si.ˈda.dɨ]
Low wordlikeness nonwords				
Cafo [ˈka.fu]	Mafopa [mɐ.ˈfo.pɐ]	Lemanado [lɨ.mɐ.ˈna.du]	Nocafozano [nɔ.kɐ.fu.zɐ̃.nʊ]	Rolinicistato [rɔ.lɨ.nɨ.siʃ.ˈta.tu]
Tuma [ˈtu.mɐ]	Dopeta [du.ˈpe.tɐ]	Dilomopa [di.lu.ˈmɔ.pɐ]	Lodanapito [lɔ.dɐ.na.ˈpi.tu]	Fatatuviricho [fɐ.tɐ.tu.vɨ.ˈɾi.ʃu]
Grapa [ˈgra.pɐ]	Gremata [grɨ.ˈma.tɐ]	Dragamato [drɐ.gɐ.ˈma.tu]	Defermicato [dɨ.fɨɾ.mɨ.ˈka.tu]	Satopogatico [sɐ.to.po.ga.ˈti.ku]
Trila [ˈtri.lɐ]	Tramafa [trɐ.ˈma.fɐ]	Trafeleste [tra.fɨ.ˈlɛʃ.tʃɨ]	Promoflicada [prɔ.mɔ.fli.ˈka.dɐ]	Cremoforosada [krε.mu.fu.ɾu.ˈza.dɐ]
